# Parasite community dynamics in an invasive vole – From focal introduction to wave front

**DOI:** 10.1016/j.ijppaw.2017.07.005

**Published:** 2017-08-04

**Authors:** Sarah E. Perkins, Tom A. White, Emily L. Pascoe, Emma L. Gillingham

**Affiliations:** a School of Biosciences, Sir Martin Evans Building, Cardiff University, Museum Avenue, Cardiff, CF10 3AX, UK; b Lancaster Environment Centre, Lancaster University, Lancaster, LA1 4YQ, UK

**Keywords:** Enemy release hypothesis, Parasite interactions, Coinfection, Invasion gradient, Lag effect, Bank vole (*Myodes glareolus*)

## Abstract

Multiple parasite species simultaneously infecting a host can interact with one another, which has the potential to influence host-parasite interactions. Invasive species typically lose members of their parasite community during the invasion process. Not only do the founding population escape their parasites, but the rapid range expansion of invaders once in the invaded range can lead to additional stochastic loss of parasites. As such, parasite community dynamics may change along an invasion gradient, with consequences for host invasion success. Here, we use the bank vole, *Myodes glareolus*, introduced as a small founding population at a point source in the Republic of Ireland in c.1920's and its ecto- and endoparasites to ask: i) how does the parasite community vary across an invasion gradient, and ii) are parasite community associations driven by host traits and/or distance from the point of host introduction? We sampled the parasite community of *M. glareolus* at the proposed focal site of introduction, at mid-wave and the invasion front, and used a parasite interactivity index and statistical models to determine the potential for the parasite community to interact. Bank voles harboured up to six different parasite taxa, with a significantly higher parasite interactivity index at the foci of introduction (*z* = 2.33, *p* = 0.02) than elsewhere, suggesting the most established parasite community has greater opportunities to interact. All but one of four synergistic parasite community associations were driven by host traits; sex and body mass. The remaining parasite-parasite associations occurred at the mid-point of the invasion wave, suggesting that specific parasite-parasite interactions are not mediated by distance from a focal point of host introduction. We propose that host traits rather than location along an invasion gradient are more likely to determine parasite-parasite interactions in the invasive bank vole.

## Highlights

• Parasite communities are more interactive in established introduction sites.
• Isolationist parasite communities are more likely towards an invasion front.
• Host traits, not location, drive parasite associations across an invasion gradient.
• A predominance of key host traits may aid invasion success.

## Graphical abstract

**Figure:**
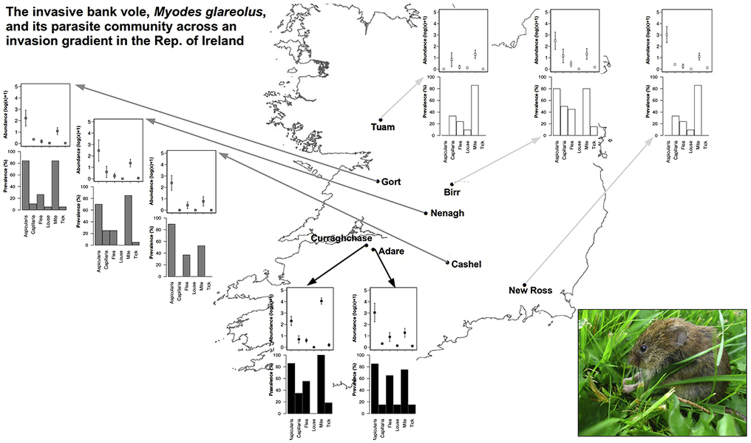

## 1 Introduction

The release of a species from the constraints of their native biotic community can give rise to changes in traits, resulting in explosive demographics within the invasive range (Colautti et al., 2004). Invasive species can be more fecund, larger in size and reach greater population sizes than their native counterparts (Blaustein et al., 1983, Keane and Crawley, 2002, Parker et al., 2013). A widely supported mechanism underlying these changed traits, and thus the success of invasive species is the enemy release hypothesis, whereby loss, or reduction of natural enemies; both predators and parasites, is translated into increased host fitness (Keane and Crawley, 2002). The regulatory effects of enemies are not equal, however. The heterogeneity of enemy release is most clearly demonstrated by parasites, as opposed to predators, where the effects upon hosts range from acute infections to almost benign (Leggett et al., 2017). Parasites can have strong regulatory effects on their host population size (Hudson et al., 1998), have positive effects on life history traits (Telfer et al., 2002), and can even be essential for maintaining biodiversity at an ecosystem level (Hudson et al., 2006).

During the invasion process there may be stochastic loss of parasite species that results in the invasive population harbouring only a subset of the parasite community of the comparable native population (Blaustein et al., 1983, Mitchell and Power, 2003, Torchin et al., 2003, Colautti et al., 2004, Phillips et al., 2010). Empirical meta-analyses have quantified that across multiple host species, the parasite diversity in the native range consists, on average, of 16 species, whilst invasive conspecifics harbour only three species of this original community (Torchin et al., 2003). As the founding population expands across an invasion gradient, the parasite community is subject to further potential parasite losses as a function of host dispersal. At the invasion front, the host population density as well as contact rates between individuals are often low, reducing the probability of parasite transmission (Morand and Poulin, 1998, Phillips et al., 2010). Additionally, stochastic fade-out of parasite species can occur due to low parasite abundance in the advancing invader, leading to some parasite species becoming locally extinct, and a ‘lag effect’, where some parasite species are absent from hosts at the invasion front (Bell and Burt, 1991, Phillips et al., 2010, Coates et al., 2017), giving them a competitive advantage.

Importantly, the parasite community within a host interacts; with antagonistic and/or synergistic interactions occurring between parasite species (Lello et al., 2004, Graham, 2008, Telfer et al., 2010). If, for example, synergistic interactions exist in the parasite community, such that the presence of one parasite species increases the abundance of another (e.g., Behnke et al., 2009, Lass et al., 2013), then, it follows that loss of one of these species in a community could lead to a reduced abundance of the other. Alternatively, if parasite-parasite interactions are antagonistic, i.e., one parasite suppresses another (e.g., Ferrari et al., 2009), loss of one of these may result in an increase of the other; a competitive release. Ultimately, the underlying mechanisms driving parasite interactions can include direct competition for resources, such as space, or indirect interactions, mediated for example by host immunity, the latter of which is linked to host traits, such as sex and body mass (Lello et al., 2013). The presence (or absence) of a given parasite species and/or the predominance of a given host trait in the host population may therefore promote a given parasite community. Whilst much work in invasion ecology has focused on the effect of parasite loss on the evolution of increased competitive ability of the host (see Strayer et al., 2006), because parasites interact with one another and the host, it is pertinent to ask how parasite loss may alter the parasite community of invasive species, and ultimately, invasion success. The first step in doing so is to determine if a parasite community interacts.

Determining whether parasites interact has been the focus of much research since Holmes's seminal papers in the 1960's (Holmes, 1961, Holmes, 1962). The methods used to determine whether particular species interact include statistical methods (e.g., Lello et al., 2004, Telfer et al., 2010) and empirical perturbation (e.g., Ferrari et al., 2009). Interactions between parasites may be erroneously detected from observational data (Fenton et al., 2010), and can be inherently difficult to detect unless empirical perturbation is used, or in the absence of perturbation, a generalised linear mixed model (GLMM) approach on longitudinal data (Fenton et al., 2014). In natural systems, however, empirical perturbation is not always possible, and longitudinal data may be logistically difficult to collect. Insight into community structure may be gained by quantifying the degree of interaction. Broadly speaking, communities can be categorised into two extreme groups; interactive vs. isolationist (Holmes and Price, 1986). Parasite communities are generally believed to lie somewhere along the interactive-to-isolationist continuum, i.e., diverse assemblages of species with high infection rates in which interspecific interactions are likely, to low diversity communities where interactions are unlikely. Qualitative indices of interactive versus isolationist communities have been proposed (e.g. Poulin and Luque, 2003), which are calculated across hosts at a population level. Ferrari et al. (2016) have recently developed an index at an individual level, giving an indication of the degree of interaction within a community and allowing comparisons to occur across multiple individuals. If parasite interactions have an evolutionary history and interspecific interactions play an important structuring role they tend to be interactive, or if interactions are unlikely, then the community is isolationist (Poulin and Luque, 2003). Across an invasion gradient, we expect parasite interactions to be greater in more established populations and weaker at the invasion front where loss of parasites, due to lag effects, may have altered community structure.

Here we ask, ‘does the parasite community, and the associations between parasites differ in an invasive species along an invasion gradient?’ and second, ‘do host traits and/or invasion location drive parasite associations?’ Using a quantitative index (see Ferrari et al., 2016) and a GLMM approach we quantified the parasite abundance, diversity and investigated the parasite community associations of the bank vole (*Myodes glareolus*), in its invaded range in the Republic of Ireland. The bank vole has a wide distribution across Europe, and has been absent from Ireland due to its isolation from Britain, which occurred during the rising sea levels at the end of the most recent glaciations (Williamson, 1996). The bank vole was, purportedly first introduced into Ireland in the 1920's on the south side of the river Shannon (Stuart et al., 2007); however the first official recording of a bank vole was in Listowel, County Kerry in 1964 (Claassens and O'Gorman, 1965). From this focal point of introduction the bank vole has spread across the Republic of Ireland at an estimated rate of 2–4 km per year (Montgomery et al., 2012, White et al., 2012). The invasive vole in the Republic of Ireland represents an excellent model system for studying enemy release with respect to parasites, because many of the predators from the native range of mainland Europe are also found in the invaded range.

## 2 Material and methods

### 2.1 Sampling along an invasion gradient

**Fig. 1:**
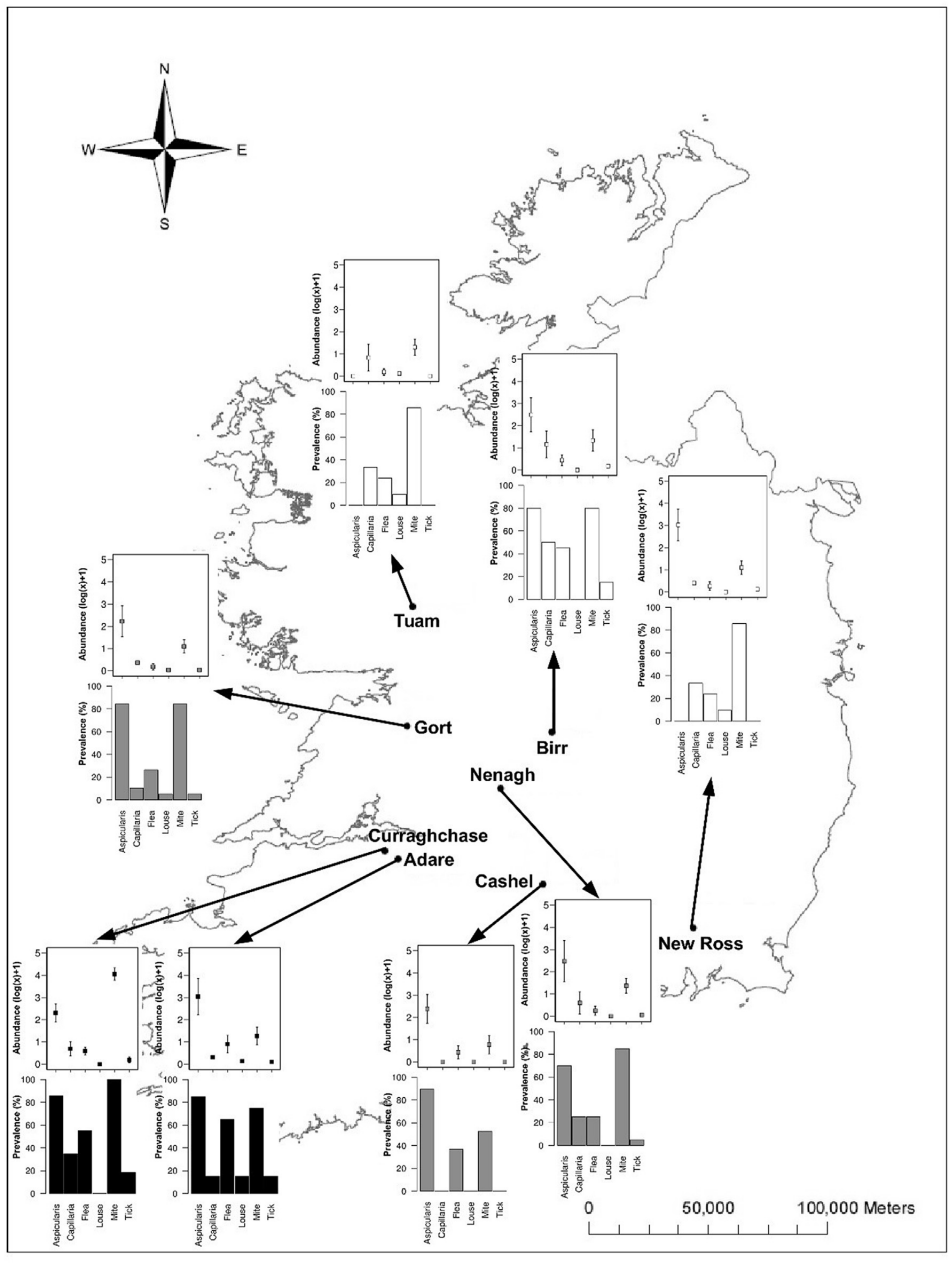
Sampling sites of the invasive bank vole, *Myodes glareolus,* across the Republic of Ireland. Curraghchase and Adare are the focal point of introduction, Gort, Nenagh and Cashel are mid-wave, and Tuam, Birr and New Ross represent the invasion front. For all trapping sites along the invasion gradient (black = focal point of introduction, grey = mid-wave, and white = invasion front), bar charts depict the prevalence of each parasite and boxplots indicate the mean parasite abundance (log *x* +1), where boxes indicate the mean, and lines the 95% confidence intervals.

Live-trapping of bank voles (*Myodes glareolus*) was carried out in suitable habitat, typically road-side verges, at three transects across an invasion gradient in the Republic of Ireland between August and December 2010. Each transect originated from the focal point of introduction of *M. glareolus*, and radiated out from there to the invasion wave front with three samples taken for each transect, so representing a historical sample, or ‘wave’ of the invasion process (Fig. 1, results). Two sites were sampled at the purported focal introduction location in County Kerry; Adare (52° 33′ 51.6780″ N, 8° 47′ 23.8380″ W) and Curraghchase (52° 37′ 1.2576″ N, 8° 52′ 39.1940″ W), three sites were sampled at a mid-point location along the invasion wave; Gort (53° 4′ 6.0600″ N, 8° 49′ 10.7828″ W), Nenagh (52° 51′ 57.8808″ N, 8° 11′ 47.7769″ W) and Cashel (52° 31′ 1.8156″ N, 7° 53′ 41.0507″ W), and a further three sites at the invasion front; Tuam (53° 30′ 55.1196″ N, 8° 51′ 14.9933″ W), Birr (53° 5′ 48.5304″ N, 7° 54′ 39.1838″ W) and New Ross (52° 23′ 45.7332″ N, 6° 56′ 43.5944″ W).

At each site, up to 50 Ugglan multiple-capture traps (Grahnab, Sweden) were set in three transects and were baited with wild bird seed and apple as a source of moisture, and contained straw for bedding. Traps were checked every 4 h and sampling continued until the first 20 bank voles of any sex had been caught at each site, except at one of the sites at the focal point of introduction, Curraghchase, where 40 animals were sampled, to ensure that each section of the invasion gradient had 60 animals sampled. Upon capture, animals were weighed and sexed, and were euthanised with a lethal dose of anaesthetic (Isofluorane, VetEquip Inc., UK) followed by cervical dislocation. All work was approved by the Ethical Committee at Cardiff University. Animals were transported to the laboratory for parasite identification.

### 2.2 Quantifying the parasite community

The gastrointestinal tract of each animal was dissected, and the parasitic helminths therein were identified to genus level (*Capillaria* and *Aspiculuris* spp.), and quantified. Broad, taxa level identification was used to provide sufficient power for model use due to low parasite abundance, and because the mechanisms by which functional groups of parasite taxa interact with one another are broadly expected to be the same across the taxa (Lello and Hussell, 2008). To quantify the ectoparasite community, parasites were removed by placing each animal on a sieve with an aperture of 63 μm under running water for 5 min. The sieve contents were examined under a stereo-microscope. We classified ectoparasites into four broad taxa at the order level; fleas, mites, ticks and lice.

To quantify the potential degree of interaction within an individuals' parasite community we calculated an ‘interactivity index’, developed by Ferrari et al. (2016). By extending the concept of ‘crowding’ previously proposed by Lloyd (1967), which measures the number of other individuals experienced by a single individual, Ferrari and colleagues translated intraspecific crowding to interspecific interactions occurring within a community. This individual-level index condenses the crowding that the parasite community experiences into a single number; an index of interactivity/isolationism, which they term an infracommunity crowding index, but hereafter we refer to as an interactivity index (*I*) (Equation (1)):

(1) *I* = 2∗∑j = 1S−1(xj∗∑i = j+1Sxi)N

where *xj* represents the abundance of the *j-th* parasite species, *S* the total number of parasite species (species richness) within the infracommunity and *N* the total parasite abundance.

We use this index because it represents an absolute measure that the community experiences into a single number, an index of interactivity/isolationism, thereby allowing for direct and meaningful comparison between samples (individuals) and sites.

### 2.3 Statistical analysis

We first examined if parasite abundance (including zeros, as defined by Bush et al., 1997) differed in sampled sites across the invasion wave. Parasite abundance was used as a response variable with position in invasion wave, host sex, host body mass, and their two-way interactions as explanatory terms. Sample site was used as a random term in a generalised linear mixed model (GLMM).

To determine if the diversity of parasites changed along the invasion wave, we calculated both the Simpson and Shannon-Weiner diversity, following (Krebs, 1999). The two diversity indices were calculated because Simpson's diversity index is able to detect differences in abundant species (calculated as 1/(∑p*i*^2^,p*i*)), where p*i* is the proportion of species *i* in the infrapopulation; whilst the Shannon-Wiener diversity index detects differences in more rare species (calculated as -∑p*i*log p*i*). Each diversity index was treated as the dependent variable in turn in a GLM, with the location along the invasion gradient included as the explanatory variable. The Simpsons' diversity index was transformed by square root, whilst Shannon-Wiener diversity index did not need to be transformed. Both models were fitted with Gaussian distributions and identity link functions.

Similarly, to determine if the degree of potential interaction in the parasite community was associated with invasion gradient location and/or host traits, we used an interactivity index (*I*) per host (after Ferrari et al., 2016) as a response variable, with invasion location, host sex, host body mass, and their two-way interactions as explanatory terms, and sample site as a random term in a GLMM, (Gaussian model with identity link). For each GLMM, backwards stepwise deletions of non-significant terms (i.e., at the *p* = 0.05 level) were used to produce the most parsimonious model. Statistical analyses were performed using the package glmmADMB, version 8.3.3 (Fournier et al., 2012, Skaug et al., 2016).

To determine if parasite community changes with location across an invasion gradient, the intensity of each taxa (excluding zeros) was treated as the dependent variable in turn and was log transformed (log (*x*+1)). Explanatory variables included in the model were: invasion wave location (focal, mid or front), host sex, host body mass, presence/absence data of each coinfecting taxa and finally, sampling site as a random term. Second order interactions were also included in the model. Fixed model terms were initially assessed by the conditional F-statistic and associated *p*-values; insignificant terms (i.e., at the *p* = 0.05 level) were removed in a step-wise manner to produce a minimal model. To assess the change in the dependent variables as predicted from the model, the predicted percentage change was calculated. This change is calculated as the percentage difference between the back-transformed dependent variable when the coinfecting taxa was absent, compared with when the coinfecting taxa was present (see Fenton et al., 2010). All GLMMs were performed in R version 3.1.2 (R Core Team, 2013), and fitted with Gaussian distributions and identity link functions.

## 3 Results

### 3.1 Does parasite abundance, and interactivity differ across an invasion gradient?

**Fig. 2:**
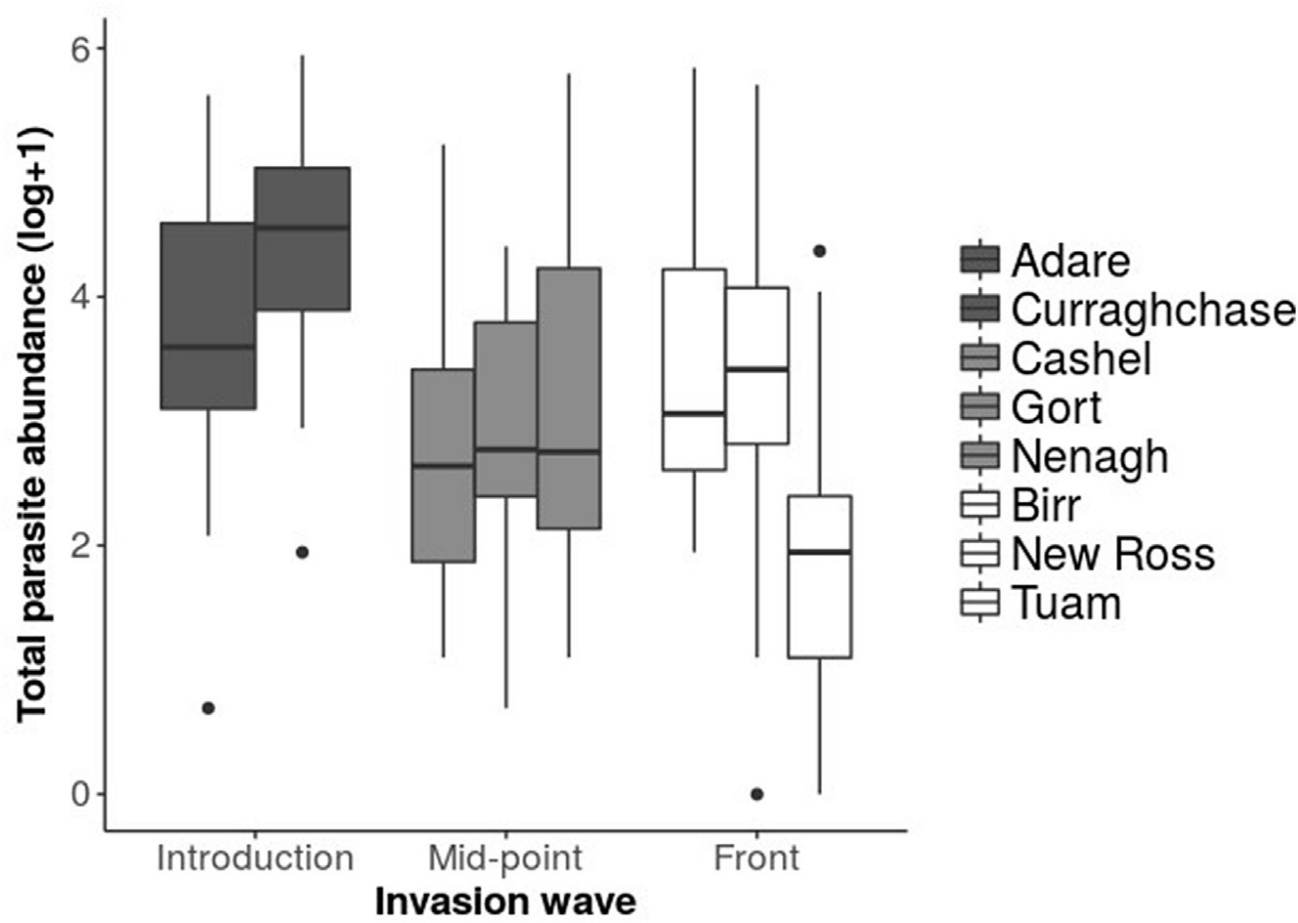
Parasite abundance of the invasive bank vole, *Myodes glareolus,* from eight locations at three points along an invasion gradient (black = focal point of introduction, grey = mid-wave, and white = invasion front) in the Republic of Ireland. Boxes represent upper and lower quartile, with median indicated, with bars representing maximum and minimum range.

Only 1.6% of all sampled hosts were parasite free, instead the majority were infected by at least one of six identified parasite taxa (98.4%), with 82.4% coinfected with more than one taxa (Fig. 1). Two genera of endoparasitic helminths were identified: *Aspiculuris* spp. and *Capillaria* spp, and ectoparasites were identified from four orders; fleas, mites, ticks and lice. Localised fade-out of parasites was seen across the invasion gradient, the prevalence of *Aspiculuris* spp., for example, was high at the focal and mid-point sites, (>70% in all locations) but at the invasion front *Aspiculuris* spp. was absent from two of the three sampled locations (Fig. 1). Parasite abundance followed a negative gradient from focal introduction to invasive front (Fig. 2), but this relationship was not significant, nor was it significantly associated with host sex, body mass or any two-way interactions of these variables. Parasite abundance at the focal site of introduction was highest, where the combined mean abundance (±S.E.) of the two sites was 106.1 ± 11.4, while the abundance at both the mid-point and invasion front were similar to each other and more than half that at the focal site of introduction (mean ± S.E. = 43.1 ± 8.8 and 43.4 ± 9.0, respectively). Parasite diversity did not significantly change across the invasion gradient, as measured by the Simpson diversity index (d.f. = 185, F = 3.03, *p* = 0.08), or the Shannon-Wiener diversity index (d.f. = 185, F = 2.43, *p* = 0.12).

**Fig. 3:**
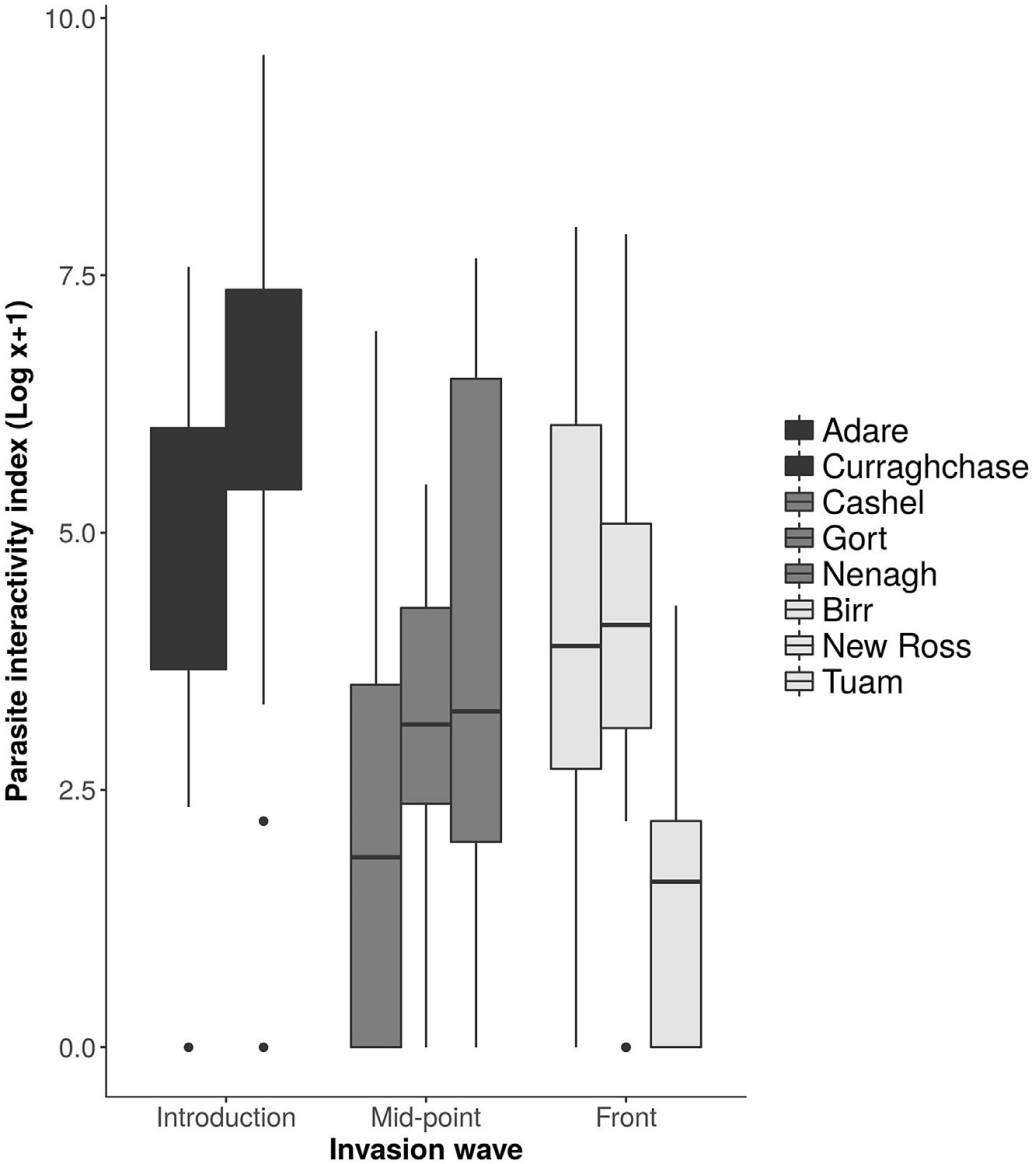
Parasite interactivity index from eight sampling sites at three points along an invasion gradient (black = focal point of introduction, grey = mid-wave, and white = invasion front) in the Republic of Ireland. Boxes represent upper and lower quartile, with median indicated, with bars representing maximum and minimum range.

The potential for the parasite community to interact was highest at the focal site of introduction; indicated by the highest interactivity index score (mean ± S.E. = 1426.0 ± 336.8), which decreased in value at the mid-point of the invasion (195.3 ± 62.7) and the invasion front (255.2 ± 82.7). Interactivity indices were not significantly different to each other at any location (introduction and mid-point: d.f. = 188, *z* = −1.90, *p* = 0.06; introduction and invasion front d.f. = 188, *z* = 0.95, *p* = 0.34; invasion front and mid-point, d.f. = 188, *z* = 0.07, *p* = 0.94; Fig. 3). Across the invasion gradient, males had a significantly lower interactivity index score than females (d.f. = 188, *z* = −2.68, *p* = 0.01), i.e., a more isolationist parasite community in males compared to females. However, males at the focal site of introduction experienced a parasite community that was significantly more likely to be interactive (i.e., higher interactive index score) than the females at this location (d.f. = 188, *z* = 2.33, *p* = 0.02).

### 3.2 Are parasites associated with host traits and/or location across an invasion gradient?

**Fig. 4:**
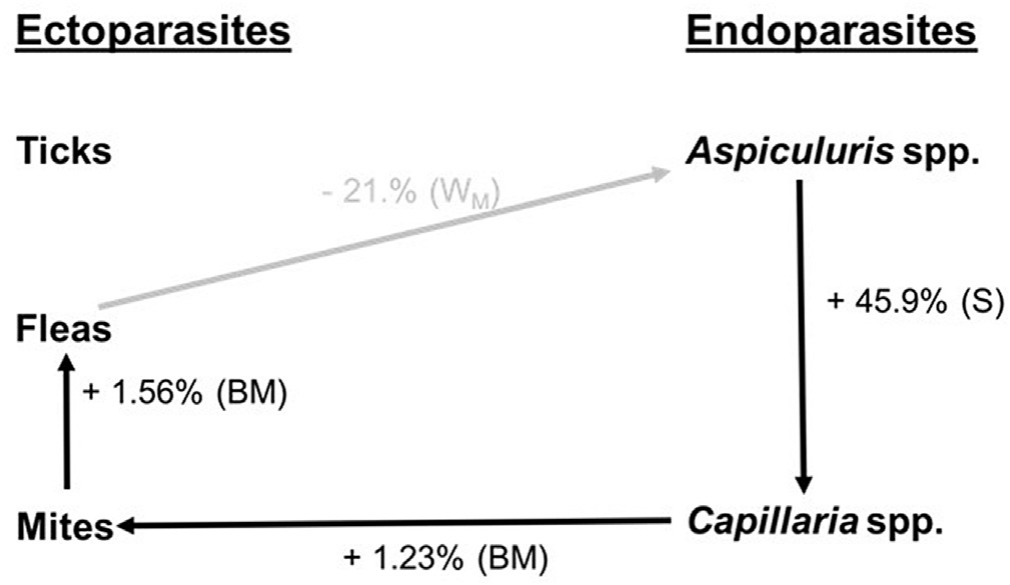
Statistical associations between ectoparasites and endoparasites infecting bank voles, *Myodes glareolus*. All associations are due to the presence of a coinfecting species, positive relationships are shown in black and negative relationships are in grey. The predicted absolute percentage change (see Methods for definition) of the dependent variable is given for each interaction. Parentheses identify whether the relationship is based on body mass (BM), host sex (S) or at the mid-point along the invasion wave (W_M_). Lice had a prevalence of less than 10% so were excluded from analyses.

Four parasite taxa were found to be associated with one another, of which three associations were host-mediated and one due to location (Fig. 4; Table 1, supplementary material). Not all parasite taxa in the community were found to potentially interact; ticks consistently were not associated with any other parasite taxa (Fig. 4). Flea abundance was negatively associated with that of *Aspiculuris* spp., and this association was driven by location along the invasion gradient. At the mid-point of the invasion gradient, individuals infected with fleas were infected with significantly fewer *Aspiculuris* spp. (backtransformed mean ± S.E. = 13.07 ± 7.22) than hosts infected with only *Aspiculuris* spp. (backtransformed mean ± S.E. = 61.03 ± 28.01). At the invasion front, individuals infected with fleas were also infected with fewer *Aspiculuris* spp., although this was not a significant interaction. Interestingly, at the focal site of introduction this association was reversed and where fleas were present *Aspiculuris* spp. intensity was higher, although not significantly so. The three host-mediated associations, driven by sex and body mass, were all synergistic, such that the presence of one taxa increased the likelihood of another. Males were infected with significantly more *Capillaria* spp. when *Aspiculuris* spp. were present (backtransformed mean ± S.E. = 14.44 ± 4.80), compared to when they were absent (backtransformed mean ± S.E. = 4.50 ± 1.56). Individuals infected with mites and *Capillaria* were of significantly higher body mass than those infected with mites only. Additionally, a positive relationship was observed between body mass and flea intensity when mites were present (Fig. 4).

## 4 Discussion

In the invasive vole population in the Rep. of Ireland, the greatest parasite abundance and highest index of parasite interactivity was at the focal point of introduction. Bank voles at the mid-wave and wave front had comparatively lower abundance and lower interactivity indices, suggesting comparatively more interactive parasite communities at the focal point of introduction, and isolationist parasite communities across the rest of the invaded range. The interactivity index was associated with host traits; males at the focal point of introduction had a parasite community that was more likely to be interactive than females, although this pattern was reversed elsewhere across the range. More detailed models examining specific parasite associations found that host traits (sex and body mass) were associated with three synergistic parasite community interactions, whilst an antagonistic interaction between parasites occurred at the mid-wave of the invasion range.

### 4.1 How does the parasite community differ across an invasion gradient?

Consistent with the enemy release hypothesis, the parasite community of the invasive bank vole was depauperate in comparison with its native counterparts. Mitochondrial DNA analysis has indicated that the bank voles present in Ireland are most closely related to those in continental Europe, in particular native bank voles within Germany (Stuart et al., 2007), for which one study found ten ecto- and endoparasite species (Klimpel et al., 2007), although only a small number of voles (n = 29) were sampled. There is no doubt that the bank voles in Ireland have lost parasite diversity, and although we used broad taxa level identification, previous species-level identification has found that bank voles in Ireland are infected with only three helminth species (Loxton et al., 2016), corroborating the parasite community observed in the present study. Consistent with the ‘lag effect’ we observed decreased parasite abundance with increasing distance from focal point or introduction to the invasion front, and stochastic loss of parasite taxa across the invasion gradient (Fig. 1). Whilst the loss of parasites may facilitate the invasion success of the bank vole, our key question was ‘how does loss of parasites within an infracommunity alter associations between parasites across the invaded range?’ Few studies have examined parasite community dynamics across invasion gradients; Diagne et al. (2016) found species richness in wild rodents (*Mus domesticus*) was higher in established sites compared with the invasion front, although they did not specifically examine how this loss of diversity may alter associations within the parasite community. It has been suggested that it is important to examine parasite community interactions in invasive species (Telfer and Bown, 2012), but to the best of our knowledge, we are the first study to have done so.

We used an index of interactivity to give an indication of the potential for a parasite community to interact, or not. The interactivity index was an order of magnitude higher at the focal site of introduction than elsewhere in the range, suggesting that the parasite community in the oldest, most established hosts has a greater opportunity to be ‘interactive’ compared to parasite communities that were comparatively more ‘isolationist’, as quantified across the rest of the invasion gradient (Fig. 4). The focal site of introduction represents a host-parasite population that has been established for almost 100 years (Stuart et al., 2007). It is to be expected that the parasite community here would have the greater potential to interact as it has had most time to establish large parasite populations and to acquire new parasites from the native biota; the former being essential to establish interactions. One parasite genus that has almost certainly been lost in the Rep. of Ireland population is *Heligmosomoides* spp. (Loxton et al., 2016). This genus is known to manipulate the immune system in order to avoid being expelled by the host (Maizels et al., 2004) and the loss of it may have had knock-on effects for the remaining parasite community.

The parasite community of males at the focal site of introduction supported a higher degree of parasite interactivity than females sampled at this location. The corollary of this observation is that males elsewhere across the invasion gradient harbour a community that may be more isolationist. Interactive communities tend to be species rich, and have many core species, whilst isolationist communities tend to have just a few species. The latter communities tend to be difficult to predict and stochastically determined (Sousa, 1994). As such, only the parasite community at our focal site was ‘predictable’ with the community elsewhere being unstable, less established and subject to stochastic loss. A more isolationist community, as observed in males as they spread throughout the invasion range, could translate into giving these individuals an advantage to establish, although it is unclear how an isolationist versus an interactive parasite community may translate into different levels of host regulation. Accompanying a reduced parasite diversity is a potential reduction in competition between parasite species for resources, potentially allowing the remaining parasite species' intensity to increase (Dobson, 1985, Silvestre et al., 2000). However, we also know that as interactivity increases, we expect other parasite traits to change, for example, to be smaller in size and less fecund (Poulin, 2007), suggesting a subsequent reduction in abundance.

### 4.2 Were parasites associated with host traits and/or location?

Of the four significant associations between the parasite community observed, there was one predicted ‘cross-boundary’ (between an ectoparasite and an endoparasite) interaction; between fleas and helminths (*Aspiculuris* spp.) in individuals at the mid-wave only, while the three other associations were driven by the biology of the host. Cross-boundary associations between parasites are unlikely to arise from competition for space, as the two parasite types occupy different parts of the host's body, or to be mediated by resource competition, although this is still possible (e.g., if both species feed on blood). The different locations occupied by these parasites (gastrointestinal system and skin) suggest that these interactions may be mediated by a systemic response of the immune system (Lello et al., 2004, Graham, 2008, Lass et al., 2013). This hypothesis requires further investigation, however, as the design of the current study allowed us only to infer associations and did not provide the opportunity to identify causal mechanisms of community interactions. Indeed, determining mechanisms underlying parasite community interactions is not straightforward, but broadly speaking, interactions may be driven by host and/or by the parasites (Lello et al., 2004, Graham, 2008).

Although the interactivity index suggests that the population at the focal site of introduction has a greater opportunity to interact, our statistical models did not find any parasite-parasite associations specific to that location to support this. One hypothesis for this mis-match is that complex communities may establish complex interactions with indirect effects and feedbacks that may only be possible to detect using the ‘gold standard’ of detecting interactions; perturbation (Fenton et al., 2014). In males only, a synergistic association between two helminths was such that where *Aspiculuris* spp. was absent or low in intensity, *Capillaria* spp. intensity was significantly reduced, suggesting males may obtain a dual benefit in reducing the abundance of both helminths if only one is lost in the invasion process. Sex is a common driver of parasite intensity (Zuk and McKean, 1996), supposedly due to the immunosuppressant effect of testosterone (Zuk and McKean, 1996, Schalk and Forbes, 1997) therefore synergistic parasite associations may offer an advantage for invasive hosts, as resources can be directed away from immunity and into other traits, such as fecundity and host size, translating into a competitive advantage.

### 4.3 Changes in the parasite community, and implications for invasion

Even forty years ago, it was noted that the bank vole was inflicting a negative impact on native biota by stripping the bark of saplings, with up to 40% of trees in one invaded area observed to be damaged by bank vole activity (Rooney and Hayden, 2002). The bank vole is not the only non-native small mammal in Rep. of Ireland. Montgomery et al. (2012) report evidence of ‘invasional meltdown’; where one non-native species facilitates introduction of another, and compounds their impact on native biota. Montgomery et al. (2012) showed that the bank vole is slowly replacing the presence of the native wood mouse, *Apodemus sylvaticus*, and together with another invasive species, the greater white-toothed shrew, *Crocidura russula,* are completely replacing the native pygmy shrew, *Sorex minutus*. Certainly, escape from regulation by parasites could be a factor that gives bank voles a competitive edge over native biota. Indeed, the authors noted that replacement of the wood mouse by the invasive bank vole reduced with distance from the focal point of introduction. In conclusion, invasive species can inflict considerable economic damage, and harm native biota. As such, understanding mechanisms that may facilitate invasion success are crucial to limiting such effects. Here, we find empirical evidence that parasite community associations in an invasive vole differ according to host traits. Future work should focus on how those associations may translate into enemy release, and so facilitate invasion success.
