# Supplementary material for: Parasite community dynamics in an invasive vole – From focal introduction to wave front
Source: Int J Parasitol Parasites Wildl. 2017 Aug 4;6(3):412–9. doi: 10.1016/j.ijppaw.2017.07.005 (PMC5715215; doi:10.1016/j.ijppaw.2017.07.005)
Supplement: Supplementary material [file mmc1.docx]

Table 1. The final statistical models of the associations between the parasite community, host traits and sample location of the invasive bank vole (*Myodes glareolus*) along an invasion gradient. Significant terms are indicated with an asterisk (*).

| **Dependent variable** | **Parameter** | **Parameter level** | **Estimate ± S.E.** | **LRT** | ***p*** |
| --- | --- | --- | --- | --- | --- |
| *Aspiculuris spp.* intensity | Intercept |  | 2.87 ± 0.35 |  |  |
|  | Wave (reference introduction) | Mid-point | -0.20 ± 0.44 | 0.84 | 0.6564 |
|  |  | Front | 0.12 ± 0.48 |  |  |
|  | Flea present/absent (reference absent) | Present | -0.27 ± 0.23 | 1.34 | 0.2463 |
|  | Wave (reference introduction) x flea present/absent (reference absent) | Mid-point x present | -1.41 ± 0.53 | 7.67 | 0.0216 * |
|  |  | Front x present | -0.71 ± 0.57 |  |  |
| *Capillaria spp. intensity* | Intercept |  | -0.87 ± 0.59 |  |  |
|  | Host sex (reference female) | Males | -0.54 ± 0.16 | 11.04 | 0.0009 * |
|  | Mass |  | 0.11 ± 0.03 | 15.14 | < 0.0001 * |
|  | *Aspiculuris* present/absent (reference absent) | Present | -0.14 ± 0.20 | 0.51 | 0.4741 |
|  | Host sex (reference female) x *Aspiculuris* spp. present/absent (reference absent) | Males x present | 0.85 ± 0.35 | 6.00 | 0.0141 * |
| Tick intensity | Intercept |  | 1.71 ± 0.73 |  |  |
|  | Mass |  | -0.04 ± 0.04 | 1.12 | 0.2905 |
| Flea intensity | Intercept |  | -3.51 ± 1.07 |  |  |
|  | Mass |  | 0.23 ± 0.05 | 1.30 | 0.2537 |
|  | Mite present/absent (reference absent) | Present | 4.69 ± 1.14 | 0.33 | 0.5667 |
|  | Mass x mite present/absent (reference absent) | Mass x present | -0.25 ± 0.06 | 16.99 | < 0.0001 * |
| Mite intensity | Intercept |  | 2.79 ± 0.60 |  |  |
|  | Mass |  | -0.02 ± 0.02 | 0.76 | 0.3828 |
|  | *Capillaria* spp. present/absent (reference absent) | Present | -0.09 ± 0.16 | 0.32 | 0.5693 |
|  | Mass x *Capillaria* spp. present/absent (reference absent) | Present | 0.11 ± 0.05 | 5.27 | 0.0217 * |
